# Chronic Metabolic Acidosis Elicits Hypertension via Upregulation of Intrarenal Angiotensin II and Induction of Oxidative Stress

**DOI:** 10.3390/antiox10010002

**Published:** 2020-12-22

**Authors:** Dinesh Aryal, Tithi Roy, Jean Christopher Chamcheu, Keith E. Jackson

**Affiliations:** 1 School of Basic Pharmaceutical and Toxicological Sciences, College of Pharmacy, University of Louisiana at Monroe, Monroe, LA 71201, USA; aryald@warhawks.ulm.edu (D.A.); royt@warhawks.ulm.edu (T.R.); chamcheu@ulm.edu (J.C.C.); 2 Department of Biomedical Affairs, Edward Via College of Osteopathic Medicine, Monroe, LA 71203, USA

**Keywords:** hypertension, metabolic acidosis, angiotensin II

## Abstract

Chronic metabolic acidosis (CMA) can be a consequence of persistent hypertension but could potentially play a role in invoking hypertension. Currently, there is a scarcity of studies examining the outcome of induced chronic acidosis on blood pressure regulation. This study investigates CMA as a cause of hypertension. Chronic acidosis was induced in Sprague Dawley rats (100–150 g) by providing a weak acid solution of 0.28 M ammonium chloride (NH_4_Cl) in tap water for 8 weeks. To determine whether the rats were acidotic, blood pH was measured, while blood pressure (BP) was monitored by tail-cuff plethysmography weekly. Rats were divided into five groups: control, CMA, CMA ± spironolactone, captopril, and tempol. Serum sodium and potassium; renal interstitial fluid (for Angiotensin II concentration); and kidney proximal tubules (for Na^+^/K^+^ ATPase- α1 concentration) were analyzed. Reactive oxygen species (ROS) were detected in renal cortical homogenates using electron paramagnetic resonance (EPR). In the CMA rats, a sustained elevation in mean arterial pressure (MAP) associated with a significant decrease in blood pH was observed compared to that of control over the 8 weeks. A significant decrease in MAP was observed in acidotic rats treated with captopril/tempol, whereas spironolactone treatment caused no decrease in MAP as compared to that of the CMA group. The interstitial angiotensin II was increased in the CMA group but decreased in the CMA with captopril and tempol groups. In addition, the urinary sodium was decreased, and the serum sodium levels increased significantly in the CMA groups as compared to that of control. However, the acidotic groups with captopril and tempol showed reduced levels of serum sodium and an elevation in urinary sodium as compared to that of the CMA group. In addition, there was a significant increase in plasma renin and no change in plasma aldosterone in the CMA group with no significant differences in plasma renin or aldosterone observed during spironolactone, captopril, or tempol treatments. The increased expression of Na^+^/K^+^ ATPase-α1 in the CMA group suggests that active transport of Na^+^ to the blood could be causative of the observed hypertension. Furthermore, the EPR analysis confirmed an elevation in superoxide (O_2_^-^) radical levels in the CMA group, but the tempol/captopril treated acidotic groups showed less (O_2_^-^) compared to that of either the CMA group or control. Taken together, our data suggest that induction of CMA could potentially be causative of hypertension, while the mechanisms underlying the increased BP could be through the activation of intrarenal Ang II and induction of oxidative stress.

## 1. Introduction

Hypertension is a chronic elevation of blood pressure that, in the long-term, causes end-organ damage and results in increased morbidity and mortality. The pathophysiology of hypertension (essential hypertension) is still unclear as various physiological mechanisms may be involved in its development. Among these mechanisms, the most extensively studied are salt intake, obesity, the renin-angiotensin system (RAS), and the sympathetic nervous system [1]. In the past few years, other factors have been investigated, including endothelial mechanisms (regulated by nitric oxide), low birth weight and imbalanced intrauterine nutrition, neurovascular anomalies, and genetics. Various epidemiological studies suggested that genetic factors account for approximately 30% of the variation in blood pressure in different populations. Some specific genetic mutations such those found in glucocorticoid-remediable aldosteronism (mutation of 11β-hydroxylase gene) and congenital adrenal hyperplasia (mutation of CYP11B1 gene) are associated with hypertension [2]. Recent studies have shifted focus to a dietary influence leading to the pathogenesis of essential hypertension. Studies have reported the association between dietary acid–base load and cardio-metabolic risk factors [3]. Disturbed blood pressure regulation has been demonstrated in several animal models with disrupted expression of acid–base transporters; and reciprocally, disturbed acid–base transport function has been described in hypertensive individuals [4]. However, it is equivocal whether there is a direct impact of altering blood pressure regulation because of the change in acid–base physiology. This study aims to investigate the effect of an induced acid–base disorder on blood pressure regulation in animals.

Metabolic acidosis is an acid–base disorder characterized by an arterial blood pH of < 7.40 and a concentration of bicarbonates [HCO3^−^] < 24 mEq/L [5]. It is a condition that occurs when the body produces excessive quantities of acid or when the kidneys are not removing enough acid from the body. Acute forms of metabolic acidosis most frequently result from the overproduction of organic acids such as keto-acids or lactic acid, whereas chronic metabolic acidosis often reflects bicarbonate wasting and/or impaired renal acidification [6]. Metabolic acidosis has been proven to be associated with a number of adverse consequences, including degradation of protein, protein-energy malnutrition, impairment of cardiovascular function, alterations of a number of endocrine functions, diminishment of glomerular filtration rate (GFR), and promotion of tubule-interstitial fibrosis [7,8,9]. There is conflicting evidence on whether acute metabolic acidosis (AMA) increases or decreases blood pressure. AMA has been shown to decrease the vascular tone and blood pressure by stimulating endothelial nitric oxide [10]. In contrast, a cross-sectional study reported that high anion gap due to the over production of organic acids in AMA could potentially elevate blood pressure [11,12]. However, it is unclear whether chronic metabolic acidosis has any significant effect on the vascular system and blood pressure regulation. Furthermore, a study demonstrated that intrarenal-RAS components (Ang II, ACE, AT1R) were upregulated during chronic metabolic acidosis [13]. Since intrarenal generation of angiotensin II plays a key role in blood pressure regulation [14], there may exist a potential linkage between chronic metabolic acidosis and blood pressure homeostasis.

The kidney is both the contributing and the target organ of the hypertensive processes [15]. Sodium and water retention in the renal tubules are associated with an increase in blood pressure. The primary cause of sodium and water retention might be an abnormal relationship between pressure and sodium excretion resulting from reduced renal blood flow, reduced nephron mass, and increased angiotensin or mineralocorticoids [1]. The kidney proximal tubule is the major site for sodium reabsorption. The sodium-electrochemical gradient created by basolateral Na^+^/K^+^ ATPase energizes the transcellular transport of multiple solutes. The changes in expression of Na^+^/K^+^ ATPase has been linked to decreased natriuresis and potential hypertension [16]. Hence, this study focused on the effect of chronic metabolic acidosis on the intrarenal RAS system and potential upregulation of Na^+^/K^+^ ATPase in regulating Na^+^ entry into the blood circulation, which would lead to volume expansion, increased arterial resistance, and eventually hypertension.

Redox signaling via reactive oxygen species (ROS) has quite recently become the focus of much attention in numerous pathological contexts, including neurodegenerative diseases, kidney disease, and cardiovascular disease. Imbalance in ROS formation and degradation has also been implicated in essential hypertension [17]. Metabolic acidosis has been shown to induce oxidative stress in the kidney that can stimulate further inflammation and fibrosis, exacerbating the damage in the failing kidney [18]. This study aims to evaluate the presence of ROS mainly (superoxides and peroxynitrites) in the renal cortex, which would have an important role in developing high blood pressure during chronic acidotic conditions.

## 2. Materials and Methods

### 2.1. Materials

Inactin (thiobutabarbital sodium), captopril, spironolactone, 4-hydroxy-Tempo (tempol), and 2- methyl butane were purchased from Sigma Aldrich Inc. Ammonium chloride was procured from Fisher Science Education. CMA 30 Linear Microdialysis Probes were obtained from CMA/Microdialysis (Harvard Apparatus, Holliston, MA, USA). Spin probing agents (CMH and CPH-hydrochoride) and DETC (diethyldithiocarbamic acid) were purchased from Enzo Life Sciences (Farmingdale, NY, USA). An Aldosterone Elisa kit was procured from Cayman Chemical, Ann Arbor, MI and a Renin Elisa kit from Sigma Aldrich, St Louis, MO. Angiotensin II Elisa kits were obtained from Sigma Aldrich Inc. Na^+^/K^+^ ATPase α1−subunit primary antibodies were procured from Cell Signaling Technology Inc. Beverly, MA, USA. Periodic acid and Schiff’s Reagent were gifted from Dr. Sharon Meyer.

### 2.2. Animals

Eight-week-old Male Sprague Dawley rats (100–150 gms) were housed at room temperature with a 12/12 light/dark cycle. Each group of animals (n = 3) were allowed to acclimatize in the animal house with free access to normal rat food and water for seven days prior to experimentation. The animal housing and experimental procedures were approved and carried out under the ethical guidelines of the Institutional Animal Care and Use Committee (IUCAC) at the University of Louisiana at Monroe (protocol no. 18OCT-KEJ-01).

### 2.3. Experimental Design

In previous studies, NH4CL in various concentrations were used to induce acidosis. We performed preliminary screening in animals with three different concentrations of ammonium chloride (0.07 M, 0.14 M, and 0.28 M) to choose a minimum concentration that would suffice to induce sustained acidosis over time. The 0.28 M concentration showed a significant decrease in blood pH as compared to that of 0.14 M, with 0.07 M showing no changes in blood pH. Since the LD50 for ammonium chloride is 1650 mg/kg/oral, we selected the 0.28 M concentration to induce chronic acidosis. Animals were divided into 5 groups (n = 3): The control group received normal tap water, the CMA group received 0.28 M ammonium chloride (NH_4_Cl) solution, and the third, fourth, and fifth groups constantly received 0.28M NH_4_Cl for 8 weeks. The third group was treated each day with spironolactone (100 mg/kg/day), the fourth group was treated with captopril (12 mg/kg/day)—an ACE inhibitor—and the fifth group was treated with tempol (100 μmol/kg/day)—a membrane-permeable free radical scavenger and metal-independent superoxide dismutase-mimetic agent that efficiently neutralizes ROS. The latter three groups were treated intraperitoneally (IP) as a single dose at the same time each day. The treatment regimen was carried out for a period of 8 weeks.

### 2.4. Blood Pressure Measurement

Animals were individually trained for seven days in a Tail-cuff Blood Pressure Analysis System (Hatteras Instruments, Cary, NC, USA) before the actual analysis of blood pressure. Blood pressure was measured each week and recorded for 8 weeks. For the in-line blood pressure (BP) measurements, the jugular vein of the anesthetized rat was catheterized and infused with physiological saline. Another catheter (PE-50, Becton Dickinson, Sparks, MD, USA) was inserted into the left carotid artery with the other end of the catheter attached to a pressure transducer (MP150 Biopack System Inc., Goleta, CA, USA). Blood pressure and heart rate were recorded using data acquisition software displayed on a computer screen in real-time.

### 2.5. pH Measurement

The urine samples from each group of animals were collected every evening from the individual cages and blood samples were collected by tail vein puncture. At the end of experiment, urine samples were collected from the exposed bladder via a suprapubic incision in anesthetized rats. Blood samples were collected in heparinized tubes from the catheterized carotid artery. The urine and blood samples were analyzed for pH every 4 weeks using an AB15 pH meter (Fisher Scientific, Waltham, MA, USA).

### 2.6. Surgical Procedure

After 8 weeks, rats from each group were weighed, anesthetized with a single dose of inactin (120 mg/kg IP), laid in a platform, and connected to an in-line blood pressure measurement system. Rats were cannulated with a trachea cannula (PE-240, Becton Dickinson, Sparks, MD, USA) through a small incision in the tracheal to ease breathing. A catheter tube was implanted in the left carotid artery with a constant supply of heparin saline solution, which was connected to the pressure transducer system for real time determination of blood pressure and heart rate. The jugular vein was catheterized and infused with normal saline (NS). NS is relatively isotonic and infused to control for volume depletion that may have occurred during arterial blood collection. It has minimal effects on altering the mean arterial pressure (MAP) as compared to the use of hypertonic saline. The urinary bladder was also catheterized for urine sample collection. The rats were positioned on their right flank, and a small left flank incision was made to expose the left kidney. CMA 30 linear microdialysis probes were inserted into the kidney cortex of the exposed kidney for renal interstitial fluid collection as previously described [19] and there after the kidney was carefully placed into the abdominal cavity, the incision was closed with a cotton ball and surgical tape. The inlet tube of the inserted probe was attached to a micro-infusion pump for physiological saline infusion (3 μL/min). The probe outlet was placed in the sample collecting tubes. After a 45-min stabilization period, blood pressure and heart rate were analyzed for 4 h. The urine, blood, and the interstitial samples were collected for further analysis.

### 2.7. Electrolyte Analysis

Urinary sodium, serum potassium, and serum sodium levels from each group of rats were analyzed utilizing an IL943 Automatic Flame Photometer (GMI Inc., Ramsey, MN, USA).

### 2.8. Interstitial Fluid Analysis

Dialysate collected from the microdialysis sampling were analyzed for Angiotensin II concentration among each group of rats using commercially available Angiotensin II ELISA kits. The competitive ELISA was performed to determine the unknown concentration of Angiotensin II in the samples from the standard curve.

### 2.9. Plasma Aldosterone and Plasma Renin Analysis

The measurement of plasma aldosterone for each group was performed according to the manufacturer’s suggestions via a commercially available aldosterone Elisa kit (Cayman Chemical, Ann Arbor, MI, USA). Similarly, plasma levels of renin were evaluated by a Renin Elisa kit (Sigma Aldrich, St Louis, MO, USA).

### 2.10. Isolation of Proximal Tubules

At the end of surgery, the kidneys from all the animals were harvested and snap frozen with 2-methylbutane and placed in −80 °C. The snap frozen kidneys were cross sectioned with cryostat (Leica 1860) and mounted on normal glass slides, each containing six sections. The slides were stained with a periodic acid-Schiff (PAS) staining procedure for isolating proximal tubules (PT) from the rest of the nephrons. The PAS staining highlighted the inner brush border membrane of the PT lumens for distinguishing PT from other nephron components. The stained slides were placed in the flat stage of a stereomicroscope (Fisher Scientific, Waltham, MA, USA) and each section was carefully punched to the distinct proximal tubules with a 0.7 mm retractable cutting cannula. The isolated PTs were collected in a tube containing 30 μL NP40 cell lysis buffer (Life Technologies, Frederick, MD, USA).

### 2.11. Protein Extraction and Western Blot Analysis

Isolated proximal tubules in a lysis buffer were vortexed, sonicated, and centrifuged, then clear supernatant was collected for Western blotting to determine the concentration of Na^+^/K^+^ ATPase α1-subunit. The protein concentration in the sample tubes were identified by bicinchoninic acid assay (BCA). The measured protein concentration in each sample was resolved by using a 4–20% gradient SDS-PAGE. After separation, proteins were transferred to 0.45 μm PVDF-Plus membranes. Membranes were blocked with 0.1% Tween −20 and 2% bovine serum albumin prior to sequential incubation with primary antisera and horse peroxidase conjugated secondary antiserum. The intensities of the blots obtained were analyzed by Image J software (ImageJ, Bethesda, MD, USA).

### 2.12. Detection of Reactive Oxygen Species by Electron Paramagnetic Resonance (EPR) Analysis

Free radicals, superoxides (O_2_-) and peroxynitrite (ONOO-) were detected using a non-destructive analytical tool, electron paramagnetic resonance spectroscopy (EPR). Because of the transient nature of the reactive oxygen species, spin probes CMH (1-hydroxy-3-methoxycarbonyl-2,2,5,5-tetramethylpyrrolidine. HCL) and CPH (1-hydroxy-3-carboxy-2,2,5,5-tetramethylpyrrolidine. HCL) were used as spin probes agents to trap O2- and ONOO-, respectively. The cortical tissue homogenates were incubated for 1 h at 37 °C in 1 mL of Krebs/HEPES buffer (pH 7.4) containing 5 μM of diethyldithiocarbamate (DETC) along with 5 mM of CMH or CPH. The samples were collected in the capillary tubes and adjusted in the sample holder of a Bruker EPR machine for analysis. The intensities were obtained and the Asc. files saved in the computer were imported to Excel for analysis. The EPR spectrometer was optimized to the following parameter settings: field sweep 100G, microwave frequency 9.87 GHz, microwave power 1.39 mW, modulation amplitude 3 G, conversion time 327.68 ms, time constant 40.96 ms, 512 points resolution, and receiver gain 1 × 10^4^.

### 2.13. Statistical Analysis

The values are presented as mean ± SEM. Statistical comparisons of the differences were performed with the use of one-way or two-way ANOVA combined with Tukey’s multiple comparison test. Some data were post analyzed with Bonferroni post hoc test. A value of *p* < 0.05 was considered statistically significant.

## 3. Results

### 3.1. Blood Pressure Measurements

**Figure 1:**
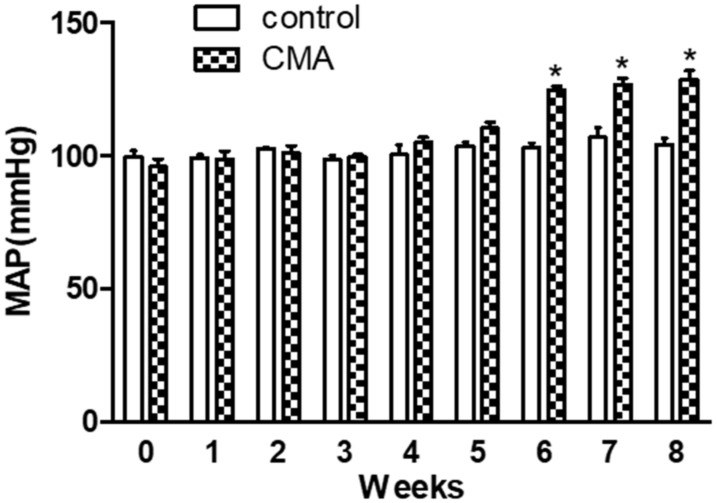
Blood pressure measurement (tail-cuff). Each bar represents values expressed as mean ± SEM. The change in mean arterial pressure (MAP) was considered significant (*) when *p* < 0.05 between two groups. The data were analyzed using two-way ANOVA followed by Bonferroni posttests.

A preliminary mean arterial pressure (MAP) difference in acidotic rats (supplied with 0.28M solution of ammonium chloride) vs. that of control (normal drinking water) rats was observed each week via tail-cuff analysis for the period of 8 weeks. There was a slight increase in mean arterial pressure (mmHg) of 110.0 ± 4.4 after 5th week in acidotic rats compared to that of control. This increase in MAP was significant (124.6 ± 3.6 mmHg) starting at the 6th week and sustained (128.46 ± 3.4 mmHg) till the 8th week (Figure 1).

### 3.2. Blood and Urine pH Measurements

**Figure 2:**
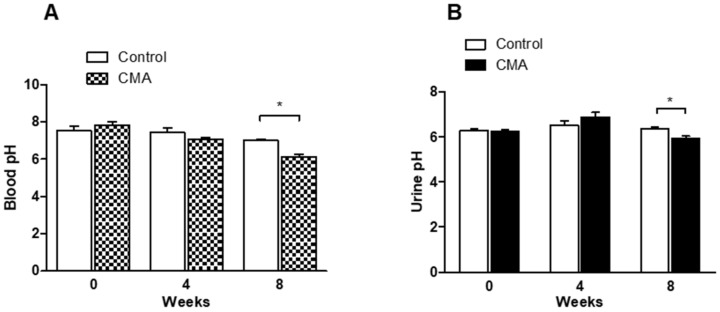
Blood (**A**) and urine (**B**) pH measurements. Each bar represents values expressed as mean ± SEM. The change in pH was considered significant (*) when *p* < 0.05 between two groups. The data were analyzed using two-way ANOVA followed by Bonferroni posttests. Measurement of blood pH (**A**) and urine pH (**B**) in control and chronic metabolic acidosis (CMA) animals.

There was no significant difference in blood and urine pH (* *p* < 0.05) at week 0 and week 4 in control vs. acidotic rats (CMA group). However, the blood pH reduced significantly to 6.13 ± 0.24 in acidotic rats compared to 7.0 ± 0.21 in control rats after 8 weeks (Figure 2A). Similarly, urine pH was also significantly decreased to 5.95 ± 0.18 in the CMA group as compared to 6.36 ± 0.22 in the control group (Figure 2B).

### 3.3. Blood Pressure Measurements for Treatment Groups

**Figure 3:**
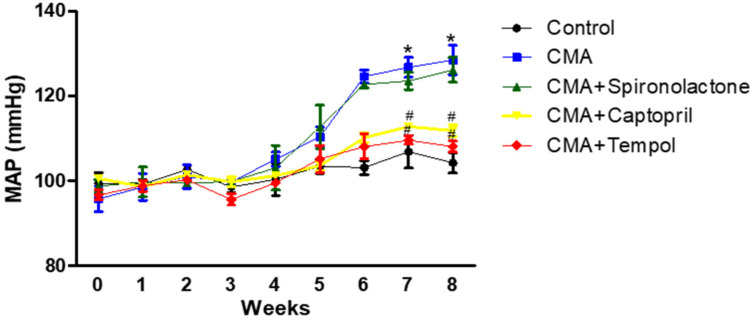
Blood pressure measurements for treatment groups (tail-cuff). Each plot represents values expressed as mean ± SEM. The change in MAP was considered significant (*) when *p* < 0.05 (control vs. CMA) groups and (^#^) *p* < 0.05 (CMA vs. CMA + captopril or CMA + tempol) groups. The data were analyzed using two-way ANOVA followed by Bonferroni multiple comparison test among all five groups.

For five different treatment groups, MAP (mmHg) was measured in each group for 8 weeks. The MAP significantly increased to 128.04 ± 4.0 mmHg in the CMA group vs. 104.07 ± 2.6 in the control group. CMA + captopril (111.83 ± 2.7) and CMA + tempol (108.08 ± 1.8) treated groups showed significant reduction in MAPs compared to that of the CMA group. Whereas CMA + spironolactone showed no significant reduction (125.71 ± 3.6) mmHg vs. that of the CMA group (Figure 3). 

### 3.4. Inline-Pressure Transducer Readings for MAP and Heart Rate

**Figure 4:**
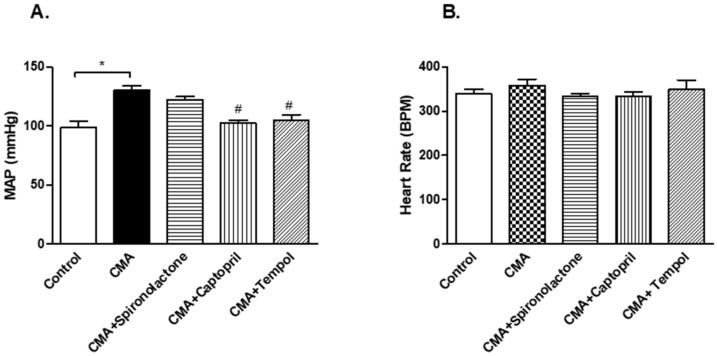
Inline-pressure transducer readings for MAP and heart rate. Each bar represents values expressed as mean ± SEM. (**A**) The change in MAP was considered significant when * *p* < 0.05 (control vs CMA) groups and ^#^ *p* < 0.05 (CMA vs. CMA + captopril or CMA + tempol) groups. (**B**) No significant changes in heart rates among the groups. The data were analyzed using one-way ANOVA followed by Tukey’s multiple comparison test among all five groups. *t*-test was performed to compare two individual groups.

The in-line MAP readings (measured in mmHg) demonstrated a significant increase in MAP of the CMA groups ((130.22 ± 6.4) vs. that of the control (98.5 ± 4.8) mmHg. The captopril and tempol treated CMA groups showed 102.48 ± 4.9 and 104.91 ± 6.2 mmHg of MAP, which is a significant reduction as compared to that of the CMA group. However, the CMA + spironolactone group showed no significant changes in MAP vs. that of the CMA group (Figure 4A). The heart rates among all five groups showed no significant changes (Figure 4B).

### 3.5. Urinary Sodium Analysis

**Figure 5:**
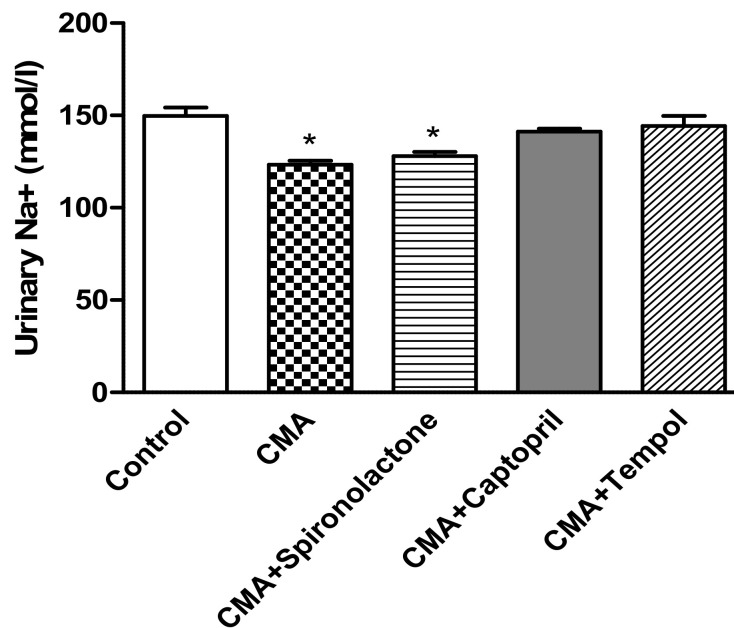
Urinary sodium analysis. Each bar represents values expressed as mean ± SEM. The change in urine sodium levels were considered significant (*) when *p* < 0.05 between groups. The data were analyzed using one-way ANOVA followed by Tukey’s multiple comparison test among all five groups.

The urinary sodium levels (mmol/L) in the CMA group (123.3 ± 3.1) and the CMA + spironolactone (127.9 ± 3.7) group were reduced significantly compared to that of the control group (149.7 ± 5.2) mmol/L. Whereas, there were no significant changes in urine sodium concentration in CMA + captopril (141.3 ± 2.8) and CMA + tempol (144.3 ± 6.3) groups vs. that of the control group (Figure 5). 

### 3.6. Serum Sodium and Potassium Levels

**Figure 6:**
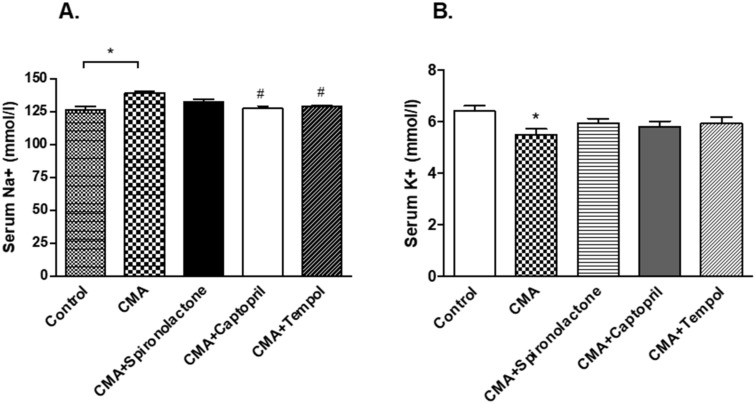
Serum sodium and potassium levels. Each bar represents values expressed as mean ± SEM. (**A**) The changes in serum sodium levels were considered significant when * *p* < 0.05 (control vs. CMA) groups and ^#^ *p* < 0.05 (CMA vs. CMA + captopril or CMA + tempol) groups. (**B**) The changes in serum potassium levels were significant when * *p* < 0.05 (Control vs. CMA) groups. The data were analyzed using one-way ANOVA followed by Tukey’s multiple comparison test among all five groups.

The serum sodium levels (mmol/L) in the CMA group (139.3 ± 1.2) were significantly (* *p* < 0.05) elevated vs. that of the control group (126.6 ± 2.1). In addition, serum sodium in the CMA + captopril and CMA + tempol groups showed significantly decreased (^#^ *p* < 0.05) values 127.5 ± 1.7 and 129.1 ± 0.8 mmol/L, respectively, vs. that of the CMA group (Figure 6A). Serum potassium on the other hand, was reduced significantly in the CMA group (5.4 ± 0.2) vs. that of the control group (6.4 ± 0.2) mmol/L. However, all other groups showed no significant changes in serum potassium levels (Figure 6B). 

### 3.7. Interstitial Fluid Analysis for Angiotensin II Concentration

**Figure 7:**
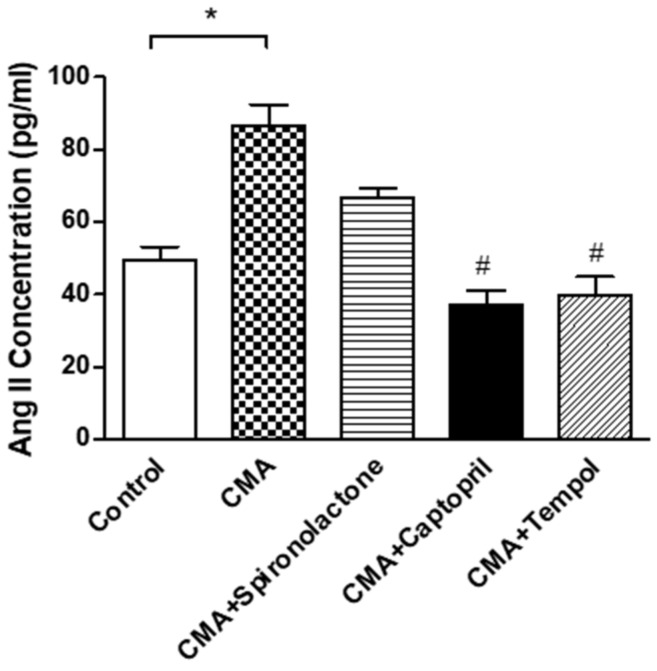
Interstitial fluid analysis for Angiotensin II concentration. Each bar represents values expressed as mean ± SEM. The changes in interstitial Ang II concentrations were considered significant when * *p* < 0.05 (control vs. CMA) groups and ^#^ *p* < 0.05 (CMA vs. CMA + captopril or CMA + tempol) groups. The data were analyzed using one-way ANOVA followed by Tukey’s multiple comparison test among all five groups.

The interstitial angiotensin II concentration (pg/mL) in the CMA group was augmented significantly (* *p* < 0.05) to 86.6 ± 5.7 vs. that of the control group (49.3 ± 3.8). In addition, there was a significant reduction in angiotensin II (^#^ *p* < 0.05) in the CMA + captopril (37.3 ± 3.7) and CMA + tempol (39.6 ± 5.2) groups when compared to that of the CMA group. The CMA + spironolactone group (66.6 ± 2.7) showed no significant changes in angiotensin II concentrations (Figure 7).

### 3.8. Plasma Aldosterone and Plasma Renin Analysis

**Figure 8:**
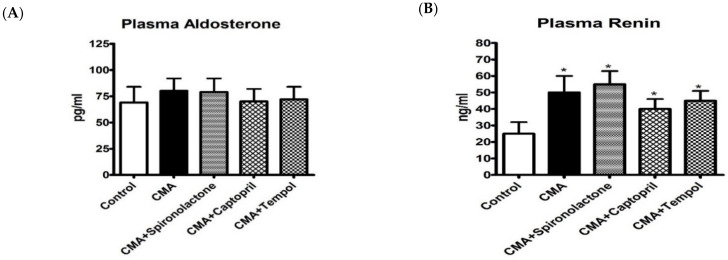
Plasma aldosterone (**A**) and plasma renin (**B**) analysis. Each bar represents values expressed as mean ± SEM. The changes in plasma aldosterone and renin were considered significant when * *p* < 0.05 (control vs. CMA) groups. The data were analyzed using one-way ANOVA followed by Tukey’s multiple comparison test among all five groups.

Plasma samples from all the groups were analyzed for changes in aldosterone and renin. There were no significant differences in plasma aldosterone concentrations (Figure 8A) in any of the groups. Plasma renin levels were significantly increased in the CMA group as compared to that of control, however, there were no significant differences in plasma renin levels in the treatment groups as compared to that of the CMA group (Figure 8B). However, there appeared to be a trend towards a decrease in plasma renin in the CMA + captopril and CMA + tempol groups (Figure 8B).

### 3.9. Periodic Acid-Schiff (PAS) Staining of Kidney Cortex Sections

**Figure 9:**
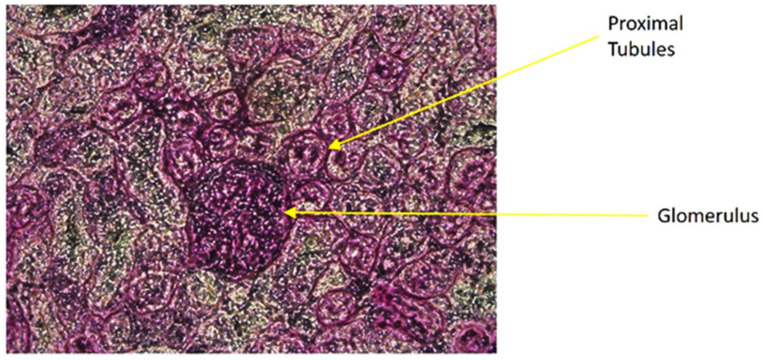
Periodic acid-Schiff (PAS) staining of kidney cortex sections.

The PAS staining of kidney cortex sections was performed as previously delineated in the methods section. The proximal tubules were identified as a thick red/pink lining of the luminal wall of the cross-sectional tube-like structure adjacent to the glomerulus in Figure 9 and isolated for analysis of transporter proteins in its membrane.

### 3.10. Protein Expression of Na^+^/K^+^ ATPase in Renal Proximal Tubule

**Figure 10:**
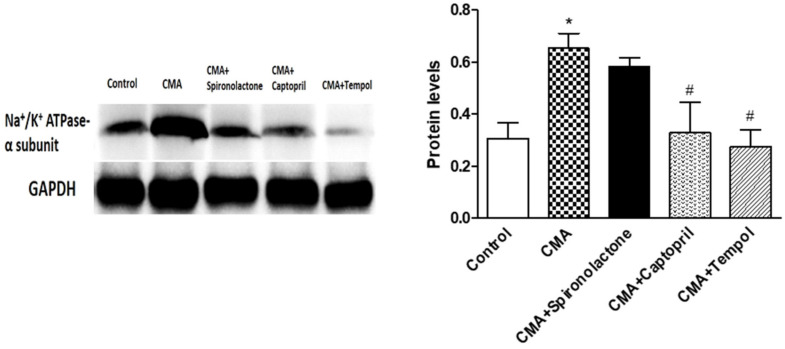
The protein expression of Na+/K+ ATPase in the renal proximal tubule. Each bar represents values expressed as mean ± SEM. The changes in protein concentrations were considered significant (*) when *p* < 0.05 (control vs. CMA) groups and (^#^) *p* < 0.05 (CMA vs. CMA + captopril or CMA + tempol) groups. The data were analyzed using one-way ANOVA followed by Tukey’s multiple comparison tests among all five groups.

The quantified densitometry intensities of protein blots showed the CMA group had a significant increase (0.64 ± 0.05) in the Na^+^/K^+^ ATPase protein levels normalized with GAPDH as compared to 0.30 ± 0.05 of the control group. Whereas the CMA + captopril and CMA + tempol groups showed significant reductions in the protein levels, 0.32 ± 0.10 and 0.27 ± 0.06, respectively, compared to that of the CMA group (Figure 10).

### 3.11. EPR Analysis for Presence of Free Radicals

**Figure 11:**
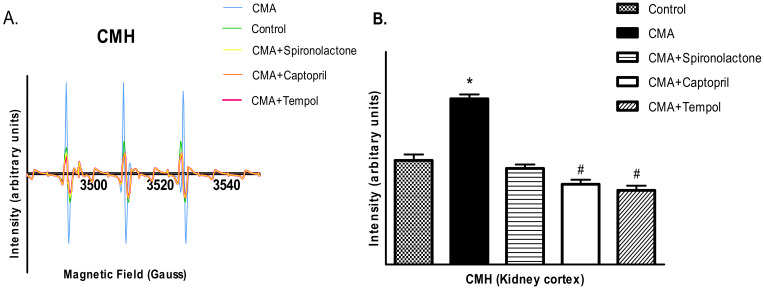
Electron paramagnetic resonance (EPR) analysis for the detection of superoxide and peroxynitrite free radicals. (**A**,**B**) show the EPR signal intensities and relative EPR signal area quantification (arbitrary units), respectively, for superoxide radicals.

**Figure 12:**
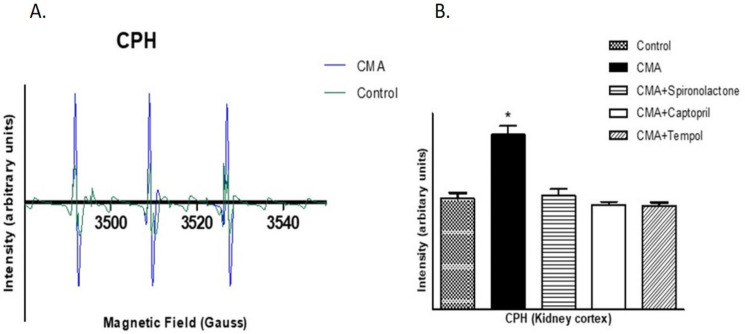
EPR analysis for peroxynitrite. (**A**,**B**) show the EPR intensities and the quantified EPR signal area of the first derivative signals, respectively, for peroxynitrite.

The CMH and CPH spin probes detected the presence of superoxide free radicals and peroxynitrite free radicals, respectively, as demonstrated by the higher intensities of EPR peaks when the renal cortical tissues were analyzed under electron paramagnetic resonance spectroscopy. The intensities of the peaks significantly increased in renal cortical tissues of the CMA group compared to that of the control group. In addition, captopril and tempol treatment reduced the intensities of the peaks (Figure 11 and Figure 12). The quantification of the peaks showed that the changes were significant.

## 4. Discussion

Several biomarkers of metabolic acidosis, including lower plasma bicarbonate, higher anion gap, and lower urinary citrate have been associated with prevalent hypertension in cross-sectional studies [20]. However, it is equivocal whether these associations represent a cause or consequence of hypertension. We sought to examine prospectively whether chemically induced chronic metabolic acidosis could elevate blood pressure, and if so, the possible mechanisms behind it.

The time frame for the development of metabolic acidosis and the underlying impact of chronic metabolic acidosis on blood pressure still remains to be elucidated. Since chronic conditions develop gradually over time and may deteriorate over an extended period of time (months to years), the current study examined these effects with the long-term development of metabolic acidosis to try to mirror the physiological disease. Previous work in the field was performed over a period of days to weeks to try to determine long term effects. Studies showed CMA induced in animals with various concentrations of ammonium chloride for 1, 3, and 7 days and 8 weeks [10,13,21]. Acidemia was developed with loss of bicarbonates in these animals, however, the body’s compensatory mechanisms such as buffer systems and the renal RAS system activated over time to play a role in minimizing acidosis [21]. Our lab performed preliminary screenings in animals with three different concentrations of ammonium chloride (0.07 M, 0.14 M, and 0.28 M) to choose a minimum concentration that would suffice to induce gradual acidosis over time. The 0.28 M concentration showed a significant decrease in blood pH in comparison to that of the 0.14 M concentration, with 0.07 M showing no changes in blood pH. Since the LD50 for ammonium chloride is 1650 mg/kg/oral, the 0.28 M concentration was used to induce acidosis. In this study we induced chronic metabolic acidosis in rats by orally treating them with a 0.28 M solution of ammonium chloride (NH_4_Cl) prepared in normal drinking water for an 8-week period. Although NH_4_Cl cannot in fact be considered an acid because it is a salt consisting of a weak base (NH_4_OH) and a strong acid (HCl), during equilibrium, NH_4_Cl dissociates to NH_3_ + HCL. HCl is a strong acid with a dissociation constant (ka) of 1 × 10^7^, which quickly dissociates into [H^+^] in plasma. The increased [H^+^] ions then consume the HCO_3_ ions in plasma to make it acidic. [NH_3_] on the other hand being a weak base with a dissociation constant (kb) of 1.80 × 10^−5^ will only partially ionize to produce ammonium cations and hydroxide anions. Therefore, a decrease in pH and the HCO_3_^-^ concentrations is expected after the NH_4_Cl administration [22]. The blood pressure measurements of the conscious rats by a tail-cuff showed an increase in the mean arterial pressure starting at week 5, and it was sustained through week 8 in the acidotic rats as compared to that of the control group. There was a decrease in blood pH at week 4, however, no significant decrease was observed until after week 5. After the 8th week, the reduction in blood pH was significant. The chronicity might have been starting to develop at week 4 and was persistent to week 8. The in-line BP measurements in the anesthetized rats confirmed that there was a sustained increase in MAP that persisted for an additional 4 h with no change in the heart rate.

It has been previously demonstrated that elevations in intrarenal angiotensin II (Ang II) cause reductions in renal function and sodium excretion that contribute to progressive hypertension and lead to renal and vascular injury. Intrarenal Ang II is not distributed in a homogenous manner but is compartmentalized in a regional and segmental manner. In the kidney cortex, Ang II is distributed in the interstitial fluid, tubular fluid, and the intracellular compartments [23]. Ang II is compartmentalized in the renal interstitial fluid and the proximal tubular compartments with much higher concentrations than those existing in systemic circulation [24]. Studies in Ang II-infused hypertensive rats have demonstrated that augmentation of intrarenal Ang II is because of the uptake of circulating Ang II via an Ang II type 1 (AT (1)) receptor-mediated internalization and also due to sustained endogenous production of Ang II [25]. A previous study showed 0.14M NH_4_Cl induced acidosis for one week, and significantly increased gene expression of renal RAS components (angiotensinogen, ACE, and AT1R), however, no changes were seen in renin/prorenin expression [13]. Taken together, the previous work investigating intrarenal production of Ang II supports the current finding that Ang II is produced intrarenally, and this production is independent to that of renin/prorenin substrate in the kidney. In the present study, the increased concentration of interstitial Ang II could be due to a mechanism other than an increase renin in the renal compartment. The reduction in interstitial Ang II levels in the captopril treated CMA rats further suggested that the increased Ang II concentrations might be because of the AT1R-mediated internalization of circulating Ang II. However, previous studies have clearly demonstrated that measured intrarenal levels of Ang II are greater than can be accounted for by circulating reuptake alone [26]. The association between (pro)renin receptor (PRR) and renal angiotensin system (RAS) has been extensively investigated but highly debated. Since its first identification from human mesangial cells, PRR was thought to be a component of the RAS based on in vitro evidence. However, subsequent animal studies were unable to prove a renin-regulatory role of PRR. In addition, overexpression of human PRR failed to affect tissue Ang II concentrations [26]. Since Ang II can be produced from different mechanisms other than renin/prorenin mediated AGT breakdown, and it can produce tubular actions independent to aldosterone secretion, there may exist a unique pathway of the intrarenal Ang II-mediated rise in blood pressure during chronic acidosis conditions. Our results further confirmed no significant changes in the plasma aldosterone levels in any of the groups, whereas there was an increase in plasma renin in CMA rats as compared to that of the control. This supported the hypothesis that chronic acidosis has no effect on systemic aldosterone release and the increase in BP is independent to the systemic aldosterone. However, the increase in plasma renin during acidosis could elevate systemic Ang II, promoting internalization; additional studies are required to examine this interesting finding. However, there were no observed significant differences in plasma renin during spironolactone, captopril, and tempol treatments, which suggests that the noted hypertension in the present study is more than likely due to the observed differences in intrarenal Ang II. Furthermore, our results revealed the reduction of urinary sodium and rise in serum sodium levels in chronically acidotic rats as compared to that of the control. This led us to examine the interstitial angiotensin II levels in both groups. The intrarenal Ang II levels is significantly high in the acidotic rats, so we deduced that intrarenal Ang II might be responsible for the increased sodium reabsorption, which could eventually increase the blood pressure. Furthermore, Ang II is a regulator of proximal tubular sodium transport, probably targeting the AT1A receptors in proximal tubules. In addition, the proximal tubule is the predominant site for sodium reabsorption [27], providing strong evidence that intrarenal Ang II may be regulating sodium reabsorption in the PT. The sodium electro-chemical gradient created by the basolateral Na^+^/K^+^ ATPase energizes the transport of ions and solutes into the peritubular capillaries [24], hence, we analyzed the samples for the expression of Na^+^/K^+^ ATPase—a transporter protein in PT cells. The results inferred that the upregulation of Na^+^/K^+^ ATPase was responsible for regulating the intrarenal Ang II-stimulated sodium reabsorption and rise in BP.

Ang II enhances tubular reabsorption either indirectly, through aldosterone stimulation via alterations in renal hemodynamics (physical factors or medullary blood flow), or by directly enhancing tubular sodium transport [28]. Therefore, we wanted to investigate whether Ang II is acting directly or indirectly. For that purpose, we treated a third group of rats with an aldosterone antagonist, spironolactone (100 mg/kg/day), and a fourth group with an ACE inhibitor, captopril (12 mg/kg/day). The BP measurements of these two groups varied in that the spironolactone-treated group showed no significant change in BP but the captopril treated group had a significantly reduced BP compared to that of the acidotic rats. Since the ACE inhibitors have been shown to reduce not only systemic but also the intrarenal Ang II levels [23,29], the treatment of captopril may have reduced the intrarenal Ang II levels, which resulted in the reduction in sodium retention and decreased the blood pressure. On the other hand, spironolactone treatment had no significant difference on BP as well as serum and urinary sodium levels compared to that of the acidotic rats, which revealed that the action of intrarenal Ang II is via direct proximal tubular sodium transport.

**Figure 13:**
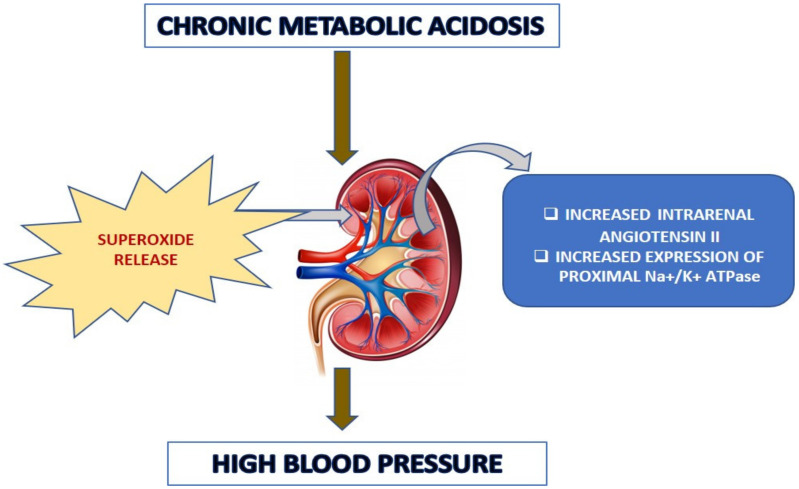
Schematic representation of possible pathophysiological routes of acidosis-induced increased blood pressure.

There are a number of pathophysiologic conditions where Ang II interacts with various local autocrine and paracrine factors (such as nitric oxide, eicosanoids, adenosine, and free radicals [30]. In this study, we wanted to determine the role of free radicals, namely superoxides and peroxynitrites, in the acidosis-induced rise in BP. For that reason, we treated the fifth group of rats with the membrane permeable superoxide dismutase (SOD) mimetic compound called 4-hydroxy-2,2,6,6-tetramethyl piperidine-1-oxyl (tempol). The BP measurements and serum and urinary sodium analyses concluded that tempol treatment reduced the BP and serum sodium levels significantly. These data provide an insight that the rise in BP in chronically acidotic rats could be the result of superoxides inactivating the vasodilator NO, which increased the systemic vascular resistance and elevated the blood pressure [31]. Surprisingly, the expression of Na^+^/K^+^ ATPase in the tempol-treated rats was significantly lower than that of the acidotic rats. This implied that the superoxides might have an effect in angiotensin II-mediated tubular transport, which needs to be clarified further. The schematic Figure 13 below demonstrates the potential pathophysiological routes for the chronic acidosis elicited hypertension.

## 5. Conclusions

Overall, the current study reported that chronic metabolic acidosis has a capacity to promote an elevation in blood pressure, and the effect could be mediated by intra-renal mechanisms involving active sodium transport in the proximal tubules and superoxide-mediated tubular transport or increased vascular resistance. These molecular mechanisms may elucidate a different origin for hypertension and may provide novel therapeutic targets for its treatment.
